# T4 genes in the marine ecosystem: studies of the T4-like cyanophages and their role in marine ecology

**DOI:** 10.1186/1743-422X-7-291

**Published:** 2010-10-28

**Authors:** Martha RJ Clokie, Andrew D Millard, Nicholas H Mann

**Affiliations:** 1 Department of Infection, Immunity and Inflammation, Maurice Shock Medical Sciences Building, University of Leicester, PO Box 138, Leicester, LE1 9HN, UK; 2 Department of Biological Sciences, University of Warwick, Gibbet Hill Road, Coventry, CV4 7AL, UK

## Abstract

From genomic sequencing it has become apparent that the marine cyanomyoviruses capable of infecting strains of unicellular cyanobacteria assigned to the genera *Synechococcus *and *Prochlorococcus *are not only morphologically similar to T4, but are also genetically related, typically sharing some 40-48 genes. The large majority of these common genes are the same in all marine cyanomyoviruses so far characterized. Given the fundamental physiological differences between marine unicellular cyanobacteria and heterotrophic hosts of T4-like phages it is not surprising that the study of cyanomyoviruses has revealed novel and fascinating facets of the phage-host relationship. One of the most interesting features of the marine cyanomyoviruses is their possession of a number of genes that are clearly of host origin such as those involved in photosynthesis, like the *psbA *gene that encodes a core component of the photosystem II reaction centre. Other host-derived genes encode enzymes involved in carbon metabolism, phosphate acquisition and ppGpp metabolism. The impact of these host-derived genes on phage fitness has still largely to be assessed and represents one of the most important topics in the study of this group of T4-like phages in the laboratory. However, these phages are also of considerable environmental significance by virtue of their impact on key contributors to oceanic primary production and the true extent and nature of this impact has still to be accurately assessed.

## Background

### The cyanomyoviruses and their hosts

**Figure 1:**
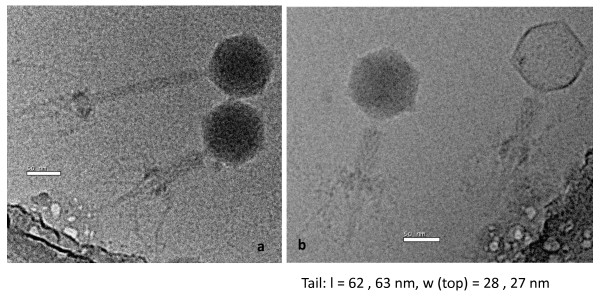
**Cryoelectron micrographs of purified S-PM2 phage particles**. (A) Showing one phage particle in the extended form and one in the contracted form both still have DNA in their heads and (B) Two phage particles with contracted tail sheaths, the particle on the left has ejected its DNA. The lack of collar structure is particularly visible in (B). The diameter of the head is 65 nm. Pictures were taken at the University of Warwick with the kind assistance of Dr Svetla Stoilova-McPhie.

**Figure 2:**
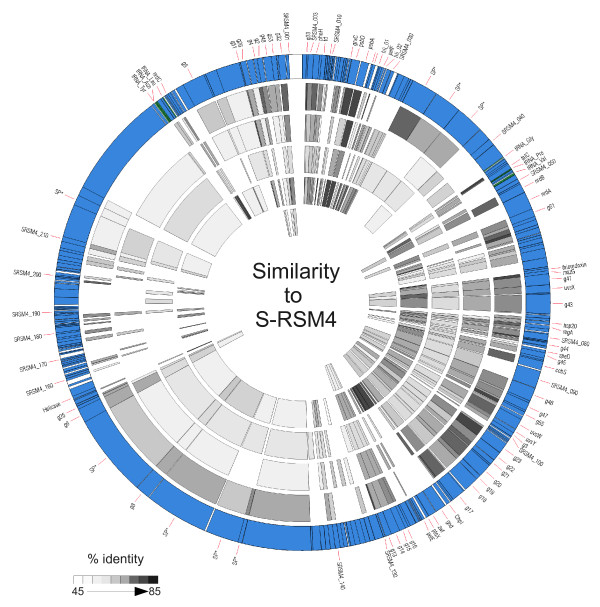
**Genome comparison of S-PM2, P-SSM2, P-SSM4, Syn9 and T4 to cyanophage S-RSM4**. The outer circle represents the genome of cyanophage S-RSM4. Genes are shaded in blue, with stop and start codon marked by black lines, tRNAs are coloured green. The inner five rings represent the genomes of S-PM2, P-SSM2, P-SSM4, Syn9 and T4 respectively. For each genome all annotated genes were compared to all genes in S-RSM4 using BLASTp and orthologues identified. The nucleotide sequence of identified orthologues were aligned and the percentage sequence identity calculated. The shading of orthologues is proportional to sequence identity, with the darker the shading proportional to higher sequence identity.

In their review on the interplay between bacterial host and T4 phage physiology, Kutter et al [1] stated that "efforts to understand the infection process and evolutionary pressures in the natural habitat(s) of T-even phages need to take into account bacterial metabolism and intracellular environments under such conditions". This statement was made around the time that the first cyanophages infecting marine cyanobacteria were being isolated and characterized and the majority of which exhibited a T4-like morphology (Figure 1) and [2-4]. Obviously, the metabolic properties and intracellular environments of obligately photoautotrophic marine cyanobacteria are very different to those of the heterotrophic bacteria that had been studied as the experimental hosts of T4-like phages and no less significant are the differences between the environments in which they are naturally found. It is not surprising, therefore, that the study of these phages has led to the recognition of remarkable new features of the phage-host relationship and this is reflected by the fact that they have been referred to as "photosynthetic phages" [5,6]. These T4-like phages of cyanobacteria have extensively been referred to as cyanomyoviruses and this is the term we have used throughout this review. Without doubt the most exciting advances have been associated with an analysis of their ecological significance, particularly with respect to their role in determining the structure of marine cyanobacterial populations and diverting fixed carbon away from higher trophic levels and into the microbial loop. Associated with this have been the extraordinary developments in our understanding of marine viral communities obtained through metagenomic approaches e.g. [7-9] and these are inextricably linked to the revelations from genomic analyses that these phages carry a significant number of genes of clearly host origin such as those involved in photosynthesis, which raises important questions regarding the metabolic function of these genes and their contribution to phage fitness. Obviously, this has major implications for horizontal gene transfer between phages, but also between hosts. Finally, from genomic sequencing it has also become apparent that the cyanomyoviruses are not only morphologically similar to T4, but are also genetically interrelated. It is still too early for these key areas, which form the major substance of this review, to have been extensively reviewed, but aspects of these topics have been covered [10-12].

Central to discussing these key aspects of cyanomyoviruses is a consideration of their hosts and the environment in which they exist. Our knowledge of marine cyanomyovirus hosts is almost exclusively confined to unicellular cyanobacteria of the genera *Synechococcus *and *Prochlorococcus*. These organisms are highly abundant in the world's oceans, and together they are thought to be responsible for 32-89% of the total primary production in oligotrophic regions of the oceans [13-15]. Although members of the two genera are very closely related to each other they exhibit major differences in their light-harvesting apparatus. Typically cyanobacteria possess macromolecular structures, phycobilisomes, that act as light-harvesting antennae composed of phycobilin-bearing phycobiliproteins (PBPs) and non-pigmented linker polypeptides. They are responsible for absorbing and transferring excitation energy to the protein-chlorophyll reaction centre complexes of PSII and PSI. Cyanobacterial PBSs are generally organised as a hemidiscoidal complex with a core structure, composed of a PBP allophycocyanin (APC), surrounded by six peripheral rods, each composed of the PBP phycocyanin (PC) closest to the core and phycoerythrin (PE) distal to the core. These PBPs, together with Chl *a*, give cyanobacteria their characteristic colouration; the blue-green colour occurs when PC is the major PBP. In marine *Synechococcus *strains, classified as sub-cluster 5.1 (previously known as marine cluster A) [16], the major light-harvesting PCB is phycoerythrin giving them a characteristic orange-red colouration. Other marine *Synechococcus *strains, more commonly isolated from coastal or estuarine waters, have phycocyanin as their major PCB and classified as sub-cluster 5.2 (previously known as marine cluster B) [16].

In contrast marine *Prochlorococcus *strains do not possess phycobilisomes and instead utilize a chlorophyll *a*_2_/*b*_2 _light-harvesting antenna complex [17]. The genetic diversity within each genus represented by a wide variety of ecotypes is thought to be an important reason for their successful colonization of the world's oceans and there is now clear evidence of spatial partitioning of individual cyanobacterial lineages at the basin and global scales [18,19]. There is also a clear partitioning of ecotypes on a vertical basis within the water column, particularly when stratification is strong e.g. [20], which at least in part may be attributable to differences in their ability to repair damage to PSII [21]. This diversity of ecotypes obviously raises questions regarding the host ranges of the cyanomyoviruses.

### Diversity

The T4-like phages are a diverse group, but are unified by their genetic and morphological similarities to T4. The cyanomyoviruses are currently the most divergent members of this group and despite clear genetic relatedness exhibit only a modest morphological similarity to the T-evens, with smaller isometric heads and tails of up to ~180 nm in length Figure 1 and [22-24], and so have been termed the ExoT-evens [22]. It has been suggested that the isometric icosahedral capsid structures of the cyanomyoviruses may reflect the fact that they only possess two (gp23 and gp20) of the five T4 capsid shell proteins with consequent effects on the lattice composition. Despite forming a discrete sub-group of the T4-like phages they exhibit considerable diversity. One study on phages isolated from the Red Sea using a *Synechococcus *host revealed a genome size range of 151-204 kb. However, the *Prochlorococcus *phage P-SSM2 is larger at 252 kb [25] and a study of uncultured viruses from Norwegian coastal waters revealed the presence of phages as large as 380 kb that could be assumed to be cyanoviruses, by virtue of their possession of the *psbA *and *psbD *genes [26].

Attempts to investigate the diversity of cyanomyoviruses began with the development of primers to detect the conserved *g20 *encoding the portal vertex protein [27] and other primer sets based on *g20 *were subsequently developed [28,29]. Diversity was found to vary both temporally and spatially in a variety of marine and freshwater environments, was as great within a sample as between oceans and was related to *Synechococcus *abundance [30-34]. With the accumulation of *g20 *sequence information from both cultured isolates and natural populations phylogenetic analysis became possible and it became apparent that were nine distinct marine clades with freshwater sequences defining a tenth [28,29,32,34-36]. Only three of the nine marine clades contained cultured representatives. Most recently a large scale survey confirmed the three marine clades with cultured representatives, but cast doubt on the other six marine clades, while at the same time identifying two novel clades [37]. The key observation from this study was that *g20 *sequences are not good predictors of a phage's host or the habitat. A substantial caveat that must be applied to these molecular diversity studies is that although the primers were designed to be specific for cyanomyoviruses there is no way of knowing whether they also target other groups of myoviruses e.g. [29].

A study employing degenerate primers against *g23*, which encodes the major capsid protein in the T4-type phages, to amplify *g23*-related sequences from a diverse range of marine environments revealed a remarkable degree of molecular variation [38]. However, sequences clearly derived from cyanomyoviruses of the Exo-Teven subgroup were only found in significant numbers from surface waters. Most recently Comeau and Krisch [39] examined *g23 *sequences obtained by PCR of marine samples coupled with those in the Global Ocean Sampling (GOS) data set. One of their key findings was that the GOS metagenome is dominated by cyanophage-like T4 phages. It is also clear from phylogenetic analysis that there is an extremely high micro-diversity of cyanomyoviruses with many closely related sequence subgroups with short branch lengths.

### Host ranges

Studies on the host range of marine cyanomyoviruses have shown wide variations. Waterbury and Valois [3] found that some of their isolates would infect as many as 10 of their 13 *Synechococcus *strains, whereas one would infect only the strain used for isolation. One myovirus isolated on a phycocyanin-rich *Synechococcus *strain, would also infect phycoerythrin-rich strains. None of the phages would infect the freshwater strain tested. Similar observations were made by Suttle and Chan [4]. A study by Millard et al., which investigated host ranges of 82 cyanomyovirus isolates showed that the host ranges were strongly influenced by the host used in the isolation process [40]. 65% of phages isolates on *Synechococcus *sp. WH7803 could infect *Synechococcus *sp. WH8103, whereas of the phages isolated on WH8103 ~91% could also infect WH7803. This may reflect a restriction-modification phenomenon. The ability to infect multiple hosts was widespread with ~77% of isolates infecting at least two distinct host strains. Another large scale study using 33 myoviruses and 25 *Synechococcus *hosts revealed a wide spread of host ranges from infection only of the host used for isolation to 17/25 hosts [41]. There was also a statistical correlation of host range with depth of isolation; cyanophage from surface stations tended to exhibited broader host ranges. A study on the host ranges of cyanophages infecting *Prochlorococcus *strains found similar wide variations in the host ranges of cyanomyoviruses, but also identified myoviruses that were capable of infecting both *Prochlorococcus *and *Synechococcus *hosts [42].

### Genetic commonalities and differences between T4-like phages from different environmental niches

The first reported genetic similarity between a cyanomyovirus and T4 was by Fuller *et al *,1998 who discovered a gene homologous to *g20 *in the cyanomyovirus S-PM2 [27]. In 2001 Hambly *et al*, then reported that it was not a single gene that was shared between S-PM2 and T4, but remarkably a 10 Kb fragment of S-PM2 contained the genes *g18*-*g23*, in a similar order to those found in T4 [22]. With the subsequent sequencing of the complete genomes of the cyanomyoviruses S-PM2[5], P-SSM4[25], P-SSM2[25], Syn9[23] and S-RSM4 [43], it has become apparent that cyanomyoviruses share a significant number of genes that are found in other T4-like phages.

### General properties of cyanophage genomes

The genomes of all sequenced cyanomyovirus are all at least 10 Kb larger than the 168 Kb of T4, with P-SMM2 the largest at 252 Kb. Genomes of cyanomyovirus have some of the largest genomes of the T4-like phages with only Aeh1 and KVP40 [44] of other T4-like phage having genomes of comparable size. The general properties of cyanophage genomes such as mol G+C content and % of genome that is coding are all very similar to that of T4 (Table 1). The number of tRNAs found within is variable, with the 2 cyanomyoviruses P-SMM2 and P-SMM4 isolated on *Prochlorococcus *having none and one respectively. In contrast the two cyanophages S-PM2 and S-RSM4 that to date are only known to infect *Synechococcus *have 12 and 25 tRNAs respectively. Previously it has been suggested a large number of tRNAs in a T4-like phage may be an adaptation to infect multiple hosts [44], this does not seem fit with the known data for cyanomyoviruses with Syn9 which is known to infect cyanobacteria from two different genera has 9 tRNAs, significantly fewer than the 25 found in S-PM2 that only infects cyanobacteria of the genus *Synechococcus*.

**Table 1:** General properties of cyanomyoviruses genomes in comparison to T4 and KVP40.

Phage	No of Genes	tRNAs	%Coding	Genome Size (Kb)	% mol G+C
T4	288	10	93	168.9	35
KVP40	386	30	92	244.8	42
S-PM2	236	25	92	196.2	37
P-SSM4	198	0	92	178.2	36
P-SSM2	330	1	94	252.4	35
Syn9	232	6	97	177.3	40
S-RSM4	238	12	94	194.4	41

Data was extracted from the genbadnk submission of each genome sequence in May 2009. T4 (accession NC_000866), KVP40 (accession number NC_005083), S-PM2 (accession number NC_006820), P-SSM4 (accession number NC006884), P-SSM2 (accession number NC006883), Syn9 (accession number NC_005083), S-RSM4 (accession number FM207411)

### Common T4-like genes

A core genome of 75 genes has previously been identified from the available T4-like genomes, excluding the cyanomyovirus genomes [25]. The cyanomyoviruses S-PM2, P-SSM4, P-SSM2 and Syn9 have been found to share 40, 45, 48 and 43, genes with T4 [5,23,25]. The majority of these genes that are common to a cyanophage and T4 are the same in all cyanomyoviruses (Figure 2).

### Transcription

Only four genes involved in transcription have been identified as core gene in T4-like phages [25]. The cyanomyoviruses are found to have three of these genes g33, g55 and *regA*. A trait common to all cyanomyoviruses is the lack of homologues to *alt*, *modA *and *modB*, that are essential in moderating the specificity of the host RNA polymerase in T4 to recognize early T4 promoters [45]. As cyanomyoviruses do not contain these genes it is thought that the expression of early phage genes may be driven by an unmodified host RNA polymerase that recognizes a σ^-70 ^factor [5]. In S-PM2 and Syn9 homologues of early T4 genes have an upstream motif that is similar to that of the σ^-70 ^promoter recognition sequence [5,23], however these have not been found in S-RSM4 (this lab, unpublished data). Cyanomyoviruses are similar to the T4-like phage RB49 in that they do not contain homologues of *motA *and *asi *which are responsible for production of a transcription factor that replaces the host σ^-70 ^factor that has been deactivated by Asi. In RB49 the middle mode of transcription is thought to be controlled by overlapping both early and late promoters [46], this is thought to be the case in S-PM2 with all homologues of T4 genes that are controlled by MotA in T4 having both an early and late promoter [5]. This also seems to be the case in Syn9 which has a number of genes that contain a number of both early and late promoters upstream [23]. However, Q-PCR was used to demonstrate that a small number of genes from S-PM2 that had middle transcription in T4, did not have a middle transcription profile in S-PM2 [46]. Subsequent global transcript profiling of S-PM2 using microarrays has suggested a pattern of transcription that is clearly different to the identified early and late patterns [Millard et al unpublished data]. Whether this pattern of transcription is comparable to the middle mode of transcription in T4 is still unknown. Furthermore, a putative promoter of middle transcription has been identified upstream of T4 middle homologues in the phage P-SMM4 and Syn9, but not in P-SSM2, S-PM2 [23] or S-RSM4 (this lab, unpublished data). Therefore, the exact mechanism of how early and middle transcription may occur in cyanomyoviruses and if there is variation in the control mechanism between cyanophage as well as difference compared to other T4-like phages is still unclear.

The control of late transcription in cyanomyoviruses and other T4 like phages seems to be far more conserved than early or middle transcription with all cyanophages sequenced to date having a homologue of *g55*, which encodes for an alternative transcription factor in T4 and is involved in the transcription of structural proteins [45]. Homologues of the T4-genes *g33 *and *g45 *which are also involved in late transcription in T4 are all found in cyanomyoviruses, but no homologues of *dsbA *(RNA polymerase binding protein) have been found. A late promoter sequence of NATAAATA has been identified in S-PM2 [5], which is very similar to the late promoter of TATAAATA that is found in T4 and KVP40 [44,45]. The motif was found upstream of a number of homologues of known T4 late genes in S-PM2 [5] and Syn9 [23]. It has since been found upstream of a number of genes in all cyanophage genomes in positions consistent of a promoter sequence [43].

### Nucleotide metabolism

Six genes involved in nucleotide metabolism are found in all cyanomyoviruses and also in the core of 75 genes found in T4-like phages [25]. The genes lacking in cyanomyoviruses from this identified core of T4-like genes are *nrdD*, *nrdG *and *nrdH*, which are involved in anaerobic nucleotide biosynthesis [45]. This is presumably as a reflection of the marine environment that cyanomyoviruses are found in, the oxygenated ocean open, where anaerobic nucleotide synthesis will not be needed. A further group of genes that are noticeable by their absence is *denA*, *ndd *and *denB*, the products of these genes are all involved in the degradation of host DNA at the start of infection [45]. The lack of homologues of these genes is not limited to cyanomyoviruses, with the marine phage KVP40 also lacking these genes [45], thus suggesting cyanomyoviruses either are less efficient at host DNA degradation [23] or that they utilise another as yet un-described method of DNA degradation.

### Replication and Repair

The replisome complex of T4 consists of the genes: *g43*, *g44*, *g62*, *g45*, *g41*, *g61 *and *g32 *are found within all cyanomyovirus genomes [5,23,25], suggesting that this part of the replisome complex is conserved between cyanomyoviruses and T4. Additionally, in T4 the genes *rnh *(RNase H) and *g30 *(DNA ligase) are also associated with the replisome complex and are involved in sealing Ozaki fragments [45] However, homologues of these genes are not found in cyanomyoviruses, with the exception of an RNase H that has been identified in S-PM2. Therefore, either the other cyanomyoviruses have distant homologues of these proteins that have not yet been identified or they do not contain them. The latter is more probable as it is known for T4 and *E*. *coli *that host DNA I polymerase and host ligase can substitute for RNase H and DNA ligase activity [45].

The core proteins involved in join-copy recombination in T4 are gp32, UvsX, UvsY, gp46 and gp47 [45], homologues of all of these proteins have been identified in all cyanomyovirus genomes [5,23,25], suggesting the method of replication is conserved between cyanomyoviruses and other T4-like phages. In the cyanomyovirus Syn9 a single theta origin of replication has been predicted [23], thus contrasting with the multiple origins of replication found in T4 [45]. The theta replication in Syn9 has been suggested to be as result of the less complex environment it inhabits compared to T4 [23]. However, as already stated it does contain all the necessary genes for recombination-dependent replication, and it is not known if other sequenced cyanomyoviruses have single theta predicted method of replication.

With cyanomyoviruses inhabiting a environment that is exposed to high-light conditions it could be assumed that the damage to DNA caused by UV would have to be continuously repaired, in T4 *denV *encodes for endonuclease V that repairs pyrimidine dimers [45], a homologue of this gene is found in the marine phage KVP40 [44], but not in any of the cyanophage genomes [5,23,25]. Given the environment in which cyanomyoviruses are found in it is likely that there is an alternative mechanism of repair, and a possible alternative has been identified in Syn9 [23]. Three genes were identified that have a conserved prolyl 4-hyroxylase domain that is a feature of the super family of 2-oxoglutarate-dependent dioxygenases, with the *E*. *coli *DNA repair protein AlkB part of this 2-oxoglutarate-dependent dioxygenase superfamily [23]. In Syn9 the genes 141, and 176 which contain the conserved domain were found to be located next adjacent to other repair enzymes UvsY and UvsX [23], this localization of these genes with other repair enzymes is not limited to Syn9 with putative homologues of these genes found adjacent to the same genes in P-SSM4. Interestingly, although putative homologues to these genes can be identified in the other cyanomyoviruses genomes they do not show the same conserved gene order.

Unlike other T4-like phages there is no evidence that any cyanomyoviruses utilize modified nucleotides such as hyroxymethyl cytosine or that they glycosylate their DNA. In addition all of the *r *genes in T4 that are known to be involved in superinfection and lysis inhibition [45] are missing in cyanophage genomes, as is the case in KVP40 [45].

### Structural Proteins

Fifteen genes have previously been identified to be conserved among T4-like phages, excluding the cyanomyoviruses, that are associated with the capsid [25] Only 9 of these genes are present within all cyanomyoviruses and other T4-like phages, whilst some of them can be found in 1 or more cyanomyoviruses. The portal vertex protein (*g24*) is absent from all cyanomyoviruses, it has been suggest that cyanomyoviruses may have an analog of the vertex protein that provides a similar function [23]. Alternatively it has been proposed that cyanomyoviruses have done away with the need for gp24 due to the slight structural alteration in gp23 subunits [39]. The proteins gp67 and gp68 are also missing from all cyanophage genomes [5,23,25], it is possible that analogs of these proteins do not occur in cyanomyoviruses as mutations in these genes in T4 have been shown to alter the structure of the T4 head from a prolate structure to that of isometric head [47,48], which is the observed morphology of cyanomyovirus heads [5,23,25]. The protein gp2, has been identified in S-PM2 [5] and S-RSM4 [43], but not any other cyanophage genomes, similarly the *hoc *gene is present only in P-SSM2, whether the other cyanomyoviruses have homologues of these genes remains unknown.

In keeping with the conservation of capsid proteins in T4-like phages, 19 proteins associated with the tail have previously been identified in T4-like phages [25], again not all these genes are present in cyanomyoviruses, those that are not include *wac*, *g10*, *g11*, *g12*, *g35*, *g34 *and *g37*. It would seem unlikely that cyanomyoviruses do not have proteins that will provide an analogous function to some of these proteins, indeed proteomic studies of S-PM2 [24] and Syn9 [23] has revealed structural proteins that have no known function yet have homologues in other cyanomyovirus genomes and therefore may account for some of these "missing" tail fiber proteins. Furthermore as new cyanomyoviruses are being isolated and characterised some of these genes may change category, for example a cyanomyovirus recently isolated from St. Kilda was shown to have distinct whiskers which we would anticipate would be encoded by a *wac *gene (Clokie unpublished observation).

### Unique cyanomyovirus genome features

The sequence of the first cyanomyovirus S-PM2 revealed an "ORFanage" region that runs from ORF 002 to ORF 078 where nearly all ORFs are all database orphans [5]. Despite the massive increase in sequence data since the publication of the genome, this observation still holds true with the vast majority of these sequences still having no similarity to sequences in the nr database. Sequences similar to some of these unique S-PM2 genes can now be found in the GOS environmental data set. The large region of database orphans in S-PM2 is similar to a large region in KVP40 that also contains its own set of ORFs that encode database orphans [44].

All cyanomyovirus genomes contain genes that are unique, with at least 65 genes identified in each cyanomyovirus that are not present in other cyanomyoviruses [43]. However, it does not appear to be a general feature of cyanomyoviruses genomes to have an "ORFanage" region as found in S-PM2. Another feature unique to one cyanomyovirus genome is the presence of 24 genes thought to be involved in LPS biosynthesis split into two clusters in the genome of P-SSM2 [49].

It has been observed for T4-like phages that there is conservation in both the content and synteny of a core T4-like genome; conserved modules such as that for the structural genes *g1-g24 *are separated by hyperplastic regions which are thought to allow phage to adapt to their host [50]. Recent analysis of the structural module in cyanomyoviruses has identified a specific region between *g15 *and *g18 *that is hyper-variable with the insertion of between 4 and 14 genes [43]. The genes within this region may allow cyanomyoviruses to adapt to their host as predicted function of these genes includes alternative plastoquinones and enzymes that may alter carbon metabolism such glucose 6-phosphate dehydrogenase and 6-phosphoglunate dehydrogenase. Whilst hyperplastic regions are found within T4-like phages the position of this hyperplastic region is unique to cyanophages.

Finally, recent work has identified CfrI, an ~225 nt antisense RNA that is expressed by S-PM2 during its infection of *Synechococcus *[51]. CfrI runs antisense to an homing endonuclease encoding gene and *psbA*, connecting these two distinct genetic elements. The function of CfrI is still unknown, however it is co-expressed with *psbA *and the homing endonuclease encoding gene and therefore thought to be involved in regulation of their expression [51]. This is the first report of an antisense RNA in T4-like phages, which is surprising given antisense transcription is well documented in eukaryotic and increasingly so in prokaryotic organisms. Although an antisense RNA has only been experimentally confirmed in S-PM2, bioinformatic predictions suggest they are present in other cyanomyovirus genomes [51].

### Signature cyanomyovirus genes

Whilst there are a large number of similarities between cyanomyoviruses and other T4-like phages as described above, and some features unique to each cyanomyovirus genome, there still remains a third category of genes that are common to cyanomyovirus but not other T4-like phages. These have previously been described as "signature cyanomyovirus genes" [25]. What constitutes a signature cyanomyovirus gene will constantly be redefined as the number of complete cyanomyovirus genomes sequenced increases. There are a number of genes common to cyanomyoviruses but not widespread or present in the T4-like super group (Table 2). Although the function of most signature cyanomyovirus genes is not known, some can be predicted as they are homologues of host genes.

**Table 2:** Shared genes in cyanomyoviruses

Functional Category	Gene	S-RSM4	S-PM2	P-SSM2	P-SSM4	Syn9	Product/Function
	*denV*	✗	✗	✗	✓	✗	Pryrimidine dimer repair
	*59^$^*	✗	✗	✓	✓	✓	ssDNA binding protein
	*rnh^$^*	✗	✓	✗	✗	✗	RNaseH
	*49^$^*	✗	✓	✓	✗	✗	Recombination endonuclease VII
	*2^$^*	✗	✓	✗	✗	✗	Protein protecting DNA ends
	*hoc*	✗	✗	✓	✗	✗	Capsid protein
	*9^$^*	✗	✗	✓	✓	✓	Baseplate socket

**Structural Proteins**	S-PM2_043	✓	✓	✓	✓	✓	structural *
	S-PM2_163	✓	✓	✓	✓	✓	structural *
	S-PM2_165	✓	✓	✓	✓	✓	structural *
	S-PM2_251	✓	✓	✓	✓	✓	structural *

**Photosynthesis**	*psbA*	✓	✓	✓	✓	✓	D2: core PSII protein
	*psbD*	✓	✓	✗	✓	✓	D1: core PSII protein
	*petE*	✓	✗	✓	✗	✓	Plastocyanin
	*petF*	✓	✗	✓	✗	✗	Ferredoxin
	*cepT*	✓	✓	✗	✗	✓	PE regulatory protein
	*ptoX*	✓	✗	✗	✓	✓	Plastoquinol terminal oxidase
	*speD*	✓	✓	✗	✓	✗	Polyamine biosynthesis
	*hli*	✓^x2^	✓^x2^	✓^x6^	✓^x4^	✓^x2^	High light inducible protein

**Carbon/phosphate metabolism**	*gnd*	✓	✗	✗	✗	✓	6-phosphogluconate dehydrogenase
	*zwf*	✓	✗	✗	✗	✓	Glucose 6-phoshate dehydrogenase
	*talC*	✓	✗	✓	✓	✓	Transaldolase
	*trx*	✓	✗	✗	✗	✓	Thioredoxin
	*phoH*	✓	✓	✓	✓	✓	Phosphate -induced stress protein
	*pstS*	✗	✗	✓	✓	✗	Phosphate -induced stress protein

**Conserved Cyanophages genes**	*mazG*	✓	✓	✓	✓	✓	Nucleoside triphosphate pyrophosphohydrolase
	*cobS*	✓	✓	✓	✓	✓	Cobalamin biosynthesis
	*prnA*	✓	✓	✓	✗	✓^x2^	Trpytophan halogenase
	S-PM2_225	✓	✓	✓	✓	✓	Oxygenase superfamily-like protein
	S-PM2_232	✓	✓	✓	✓	✓	Putative Helicase
	S-PM2_113	✓	✓	✓	✓	✓	Unknown
	S-PM2_117	✓	✓	✓	✓	✓	Unknown
	S-PM2_119	✓	✓	✓	✓	✓	Unknown
	S-PM2_138	✓	✓	✓	✓	✓	Unknown
	S-PM2_141	✓	✓	✓	✓	✓	Unknown
	S-PM2_164	✓	✓	✓	✓	✓	Unknown
	S-PM2_056	✓	✓	✓	✓	✓	Unknown
	S-PM2_186	✓	✓	✓	✓	✓	Unknown
	S-PM2_187	✓	✓	✓	✓	✓	Unknown
	S-PM2_194	✓	✓	✓	✓	✓	Unknown
	S-PM2_198	✓	✓	✓	✓	✓	Unknown

The table was modified from [25,45]. Genes were called present (#10003;) or absent (#10007) using previous annotations [5,23,25] and BLASTp with a cut off value of <10-5.

* Genes previously identified as structural proteins by mass spectrometry [23,24].

^$ ^Genes previously identified as core to T4-like phages [25].

The most obvious of these is the collection of genes that are involved in altering or maintaining photosynthetic function of the host. The most well studied and first discovered gene is the photosynthetic gene *psbA *which was found in S-PM2 [52], since then this gene has be found in all complete cyanomyovirus genomes [5,23,25]. The closely associated gene *psbD*, is found in all completely sequenced cyanomyovirus genomes with the exception of P-SSM2 [25]. However this is not a universal signature as although one study using PCR has found *psbA *to present in all cyanomyovirus isolates tested [49] or a different study showed that it was only present in 54% cyanomyoviruses [53]. The presence of *psbD *in cyanomyoviruses appears to be linked to the host of the cyanomyovirus with 25% of 12 phage isolated on *Prochlorococcus *and 85% of 20 phage isolated on *Synechococcus *having *psbD *[53]. With the most recent study using a microarray for comparative genomic hybridisations, found 14 cyanomyoviruses, known to infect only *Synechococcus*, contained both *psbA *and *psbD *[43]. *psbA *and *psbD *have also been detected in a large number of environmental samples from subtropical gyres to Norwegian coastal waters [26,54,55]. With cyanomyovirus derived *psbA *transcripts being detected during infection in both culture [56] and in the environment [57].

In summary, both *psbA *and *psbD *are widespread in cyanomyovirus isolates and that *psbD *is only present if *psbA *is also present [49,53] and cyanomyovirus are thought to have gained these genes on multiple occasions independently of each other [46,49,53].

In addition to *psbA *and *psbD*, other genes not normally found in phage genomes have been identified, these include *hli, cobS, hsp *that are found in all complete cyanomyovirus genomes. Additionally the genes *petE, petF*, *pebA*, *speD*, *pcyA*, *prnA*, *talC*, *mazG*, *pstS*, *ptoX, cepT*, and *phoH *have all been found in at least one or more cyanomyovirus genomes. In addition to being found in complete phage genomes these accessory genes have been identified in metagenomic libraries [54,55]. Not only are these genes present in the metagenomic libraries they are extremely abundant; e.g. there were 600 sequences homologous to *talC *in the GOS data set, in comparison there were 2172 sequences homologous to a major capsid protein [55]. The metabolic implications of these genes are discussed in the next section.

### Cyanomyovirus-like sequences in metagenomes

In the last few years there has been a massive increase in the sequence data from metagenomic studies. The Sorcerer II Global Ocean Expedition (GOS) alone has produced 6.3 billion bp of metagenomic data from various Ocean sites [58], with the viral fraction of the metagenome dominated by phage like sequences [55]. Subsequent analysis by comparison of these single reads against complete genomes allows, recruitment analysis, allows identification of genomes that are common in the environment. In the GOS data set, only the reference genome of P-SSM4 was dominant [55].

A further study that examined 68 sampling sites, representative of the four major marine regions, showed the wide spread distribution of T4-like cyanomyovirus sequences in all four major biomes [7]. With increased cyanomyovirus sequences in the Sargasso Sea biome compared to the other regions examined [7]. In a metagenomic study of the viral population in the Chesapeake Bay the viral population was dominated by the *Caudovirales*, with 92% of the sequences that could be classified falling within this broad group [8]. A finer examination of this huge data set revealed that 13.6% and 11.2% of all homologues identified were against genes in the cyanomyovirus P-SSM2 and P-SSM4 respectively [8].

Even in metagenomic studies that have not specifically focused on viruses, cyanomyovirus sequences have been found. For example, in a metagenomic study of a subtropical gyre in the Pacific, up to 10% of fosmid clones contained cyanophages-like sequences, with a peak in cyanophages-like sequences at a depth of 70 m, which correlated with the maximal virus:host ratio [54]. All of the metagenomic studies to date have demonstrated the widespread distribution of cyanomyovirus like sequences in the ocean and provided a huge reservoir of sequence from the putative cyanomyovirus pan-genome. However, with only five sequenced cyanomyovirus it is not known how large the pan-genome of cyanomyoviruses really is. With every newly sequenced cyanomyovirus genome there has been ~25% of total genes in an individual phage that are not found in other cyanomyoviruses. Even for core T4-like genes their full diversity has probably not been discovered. By examining the diversity of ~1,400 gp23 sequences from the GOS data set it was observed that the cyanomyovirus-like sequences are extremely divergent and deep branching[39]. It was further concluded that diversity of T4-like phages in the world's Oceans is still to be fully delimited [39].

### Metabolic Implications of unique cyanomyovirus genes

#### Cyanomyoviruses and Photosynthesis

Cyanomyoviruses are unique among T4-like phages in that their hosts utilize light as their primary energy source; therefore it is not to surprising cyanomyoviruses carry genes that may alter the photosynthetic capability of their hosts. The most well studied of the photosynthetic phage genes are *psbA *and *psbD*, which encode for the proteins D1 and D2 respectively. The D1 and D2 proteins form a hetero-dimer at the core of photosystem II (PSII) where they bind pigments and other cofactors that ultimately result in the production of an oxidant that is strong enough to remove electrons from water. As an unavoidable consequence of photosynthesis there is photo-damage to D1 and to a lesser extent the D2 protein, therefore all oxygenic photosynthetic organisms have evolved a repair cycle for PSII [59]. The repair cycle involves the degradation and removal of damage D1 peptides, and replacement with newly synthesized D1 peptides [59]. If the rate of removal and repair is exceeded by the rate of damage then photoinhibiton occurs with a loss of photochemical efficiency in PSII [60]. A common strategy of T4-like phages is to shutdown the expression of host genes after infection, but if this was to occur in cyanomyoviruses then there would be a reduction in the reduction efficiency of the PSII repair cycle and thus reduced photosynthetic efficiency of the host. This would be detrimental to the replication of phage and it has therefore been proposed that cyanomyoviruses carry their own copies of *psbA *to maintain the D1 repair cycle [52]. There is strong evidence to suggest that this is the case with Q-PCR data proving the *psbA *gene is expressed during the infection cycle for the phage S-PM2 and that there is no loss in photosynthetic efficiency during the infection cycle [56]. Further evidence for the function of these genes can be gained from P-SSP7 a podovirus that also express *psbA *during infection with phage derived D1 peptides also being detected in infected cells [61]. Although as yet phage mutants lacking these genes have yet to be constructed the results of modelling with in silico mutants suggests that *psbA *is a non essential gene [62] and that its fitness advantage is greater under higher irradiance levels [62,63]

The carriage of *psbD *is assumed to be for the same reason in the maintenance of photosynthetic efficiency during infection, indeed it has been shown that *psbD *is also expressed during the infection cycle [Millard et al unpublished data]. However, not all phage are known to carry both *psbD *and psbA, in general that the broader the host range of the phage the more likely it is to carry both genes[40,49]. It has therefore been suggested that by carrying both of these genes that phage can ensure the formation of a fully functional phage D1:D2 heterodimer [49].

Cyanomyoviruses may maintain the reaction centres of their host in additional and/or alternative ways to the replacement of D1 and D2 peptides. The reaction centre of PSII may also be stabilized by *speD *a gene that has been found in S-PM2, P-SSM4 and S-RMS4. *speD *encodes S-adenosylmethionine decarboxylase a key enzyme in the synthesis of the polyamines spermidine and spermine. With polyamines implicated in the stabilising the *psbA *mRNA in the cyanobacterium *Synechocystis *[64], altering structure of PSII [65] and restoring photosynthetic efficiency [66], it has been proposed they also act to maintain the function of the host photosystem during infection [11].

Whilst *psbA *and *psbD *are the most studied genes that may alter photosynthetic ability, they are certainly not the only genes. The carriage of *hli *genes that encode high light inducible proteins (HLIP) are also thought to allow the phages host to maintain photosynthetic efficiency under different environmental conditions. HLIP proteins are related to the chlorophyll *a/b*-binding proteins of plants and are known to be critical for allowing a freshwater cyanobacteria *Synechocysti*s to adapt to high-light conditions [67]. The exact function in cyanomyoviruses is still unknown, they probably provide the same function of as HLIPs in their hosts, although this function is still to be fully determined. It is apparent that the number of *hli *genes in phage genome is linked to the host of the cyanomyovirus with phage that were isolated on *Prochlorococcus *(P-SSM2 & P-SSM4) having double the number of *hli *genes found on the those phage isolated on *Synechococcus *(S-RSM4, Syn9, S-PM2) (Table 2). The phylogeny of these genes suggest that some of these *hli *genes are *Prochlorococcus *specific [68], probably allowing adaptation to a specific host.

A further photosynthetic gene that may be advantageous to infection of a specific host is *cepT*. S-PM2 was the first phage found to carry a *cepT *gene [5], it is also now found in Syn9 [23], S-RSM4 and 10 other phages infecting *Synechococcus *[43], but is not found in the phage P-SSM2 and P-SSM4 which were isolated on *Prochlorococcus *[49]. *cepT *is thought to be involved in regulating the expression of phycoerythrin (PE) biosynthesis [69], PE is a phycobiliprotein that forms part of the phycobilisome that is responsible for light-harvesting in cyanobacteria [70], the phycobilisome complex allows adaptation to variable light conditions such as increased UV stress [70]. Recently it has been shown that amount of PE and chlorophyll increases per cell when the phage S-PM2 infects its host *Synechococcus *WH7803, with this increases in light harvesting capacity thought to be driven by the phage to provide enough energy for replication [6] with phage *cpeT *gene responsible for regulation of this increase [71]. As *Prochlorococcus *do not contain a phycobilisome complex that contains PE, which the *cpeT *regulates expression of, it is possibly a gene advantageous to cyanomyoviruses infecting *Synechococcus*.

Phage genes involved in bilin synthesis are not limited to *cepT*, within P-SSM2 the bilin reductase genes *pebA *and *pcyA *have been found and are expressed during infection [72]. The *pebA *gene is functional *in vitro *and catalyses a reaction that normally requires two host genes (*pebA *&*pebB*) and has since being renamed *pebS*, this single gene has been suggested to provide the phage with short tern efficiency over long term flexibility of the two host genes [72]. Despite evidence of expression and that the products are functional it is unclear how these genes are advantageous to cyanomyoviruses infecting *Prochlorococcus *which do not contain standard phycobilisome complexes.

Alteration of host photosynthetic machinery appears to be of prime importance to cyanomyoviruses with a number of genes that may alter photosynthetic function. In addition to maintaining PSII centres and altering bilin synthesis, a further mechanism for diverting the flow of electrons during photosynthesis may occur. A plastoquinol terminal oxidase (PTOX)-encoding gene was first discovered in P-SMM4 [25] and then in Syn9 [23] and more recently has been found to be widespread in cyanomyoviruses infecting *Synechococcus*. The role of PTOX in cyanobacteria, let alone cyanomyoviruses, is not completely understood, but it is thought to play a role in photo-protection. In *Synechococcus *it has been found that under iron-limited conditions CO_2 _fixation is saturated at low light intensities, yet the reaction centres of PSII remain open at far higher light intensities. This suggests an alternative flow of electrons to receptors other than CO_2 _and the most likely candidate acceptor is PTOX [73]. The alternative electron flow eases the excitation pressure on PSII by the reduction of oxygen and thus prevents damage by allowing an alternative flow of electrons from PSII [73]. Further intrigue to this story in that PTOX encoding genes are not present in all cyanobacterial genomes and are far more common in *Prochlorococcus *genomes than in *Synechococcus *genomes. Therefore, phage may not only maintain the current *status quo *of the cell as in the same manner *psbA *is thought to, but may offer an alternative pathway of electron flow if its host does not carry its own PTOX genes. Although this is speculative it is already known that cyanomyoviruses that carry PTOX genes can infect and replicate in *Synechococcus *WH7803 that does not have PTOX-encoding gene of its own.

#### Carbon Metabolism

All sequenced cyanomyoviruses have genes that may alter carbon metabolism in their hosts, although not all cyanomyoviruses have the same complement of genes [5,23,25]. Syn9 [23] and S-RSM4 have *zwf *and *gnd *genes encoding the enzymes glucose 6-phosphate dehydrogenase (G6PD) and 6-phosphogluconate dehydrogenase which are enzymes utilised in the oxidative stage of the pentose phosphate pathway (PPP). The rate-limiting step in the PPP is the conversion of glucose-6-phosphate, which is catalysed by G6PD. It could be advantageous for a phage to remove this rate-limiting step in order to increase the amount of NADPH or ribulose 5-phosphate it requires for replication. Whether the phage removes this rate limitation by encoding a G6PD that is more efficient than the host G6PD or simply producing more, is not known. Without experimental data the proposed advantages of these genes are speculative.

There are at least 5 modes in which the PPP can operate depending on the requirements of the cell [74]. It might be assumed that for a phage the priority might be to produce enough DNA and protein for replication, thus use the mode of PPP that produces more ribulose 5- phosphate at the expense of NAPH. The production of ribulose 5-phosphate could then be used as the precursors for nucleotide synthesis. This mode of flux would result in the majority of glucose-6-phosphate being converted to fructose-6-phosphate and glyceraldehyde 3-phosphate. These molecules could then be converted to ribulose 5-phosphate by a transaldolase and transketolase.

Therefore, it is not surprising that *talC *has been detected in four of the five sequenced cyanomyovirus genomes, in viral metagenomic libraries [54], and in fragments of cyanomyovirus genomes S-BM4 [53] and SWHM1 (this lab unpublished data). *talC *encodes a transaldolase, an important enzyme in linking PPP and glycolysis, that if functional would catalyze the transfer of dihydroxyacetone from fructose 6-phospate to erythrose 4-phosphate, giving sedoheptulose 7-phosphate and glyceraldehyde 3-phosphate. However, currently this alteration of the PPP is speculation as other modes of flux are just as possible depending on the circumstances the phage find it self within its host with alternative modes leading to an increase in the production ATP and NADPH [23].

It does appear that maintaining or altering carbon metabolism is important to cyanomyoviruses as the genes *trx *is also found Syn9 and S-RSM4. The product of *trx *is thioredoxin, an important regulatory protein that is essential in the co-ordination of the light-dark reactions of photosynthesis by the activation of a number of enzymes, one of the few enzymes that it suppresses is glucose-6-phosphate dehydrogenase [75]. The reduced form of thioredoxin controls enzyme activity, with thioredoxin itself reduced by ferredoxin in a process catalysed by ferredoxin-thioredoxin reductase [76]. Whilst no cyanomyovirus have been found to have ferredoxin-thioredoxin reductase, the cyanomyovirus S-RSM4 and P-SSM4 do have *petF*, that encodes ferredoxin,. Ferredoxin acts as an electron transporter which is associated with PSI, whether the phage petF replaces host petF function is not known.

The function of another electron transporter is also unclear, some cyanophages (S-RSM4, Syn9, P-SSM2) have a homologue of *petE*. Host *petE *encodes plastocyanin, which transfers electrons from the cytochrome *b*_6_*f *complex of photosystem II to P700^+ ^of photosystem I. It is known cyanobacterial *petE *mutants show both a reduced photosynthetic capacity for electron transport and slower growth rate [77]. Thus, it is possible that the phage *petE *is beneficial by means of maintaining photosynthetic function.

Whilst there are a number of genes, *trx, zwf*, *gnd*, *petE*, *petF *that may alter host carbon metabolism, unravelling their function is not a trivial task, this is exemplified genes such as *trx *that can regulate enzymes in the Calvin cycle, PPP, and gluconeogenesis. This is further complicated by the fact that to date no two cyanomyovirus to date have exactly the same complement of genes that may alter carbon metabolism, with S-PM2 having none of the above mentioned and at the opposite end of the spectrum S-RSM4 has the full complement. However, the widespread distribution of these genes in cyanomyoviruses suggests their presence is not coincidental and they may be advantageous to cyanomyovirus under certain environmental conditions.

#### Phosphate Metabolism

The gene *phoH *has been found in all sequenced cyanomyovirus genomes, and in KVP40 [44]. The function of the gene in cyanomyovirus is not known; in *E. coli *it is known that *phoH *forms part of the pho regulon, with *phoH *regulated by *phoB *with increased expression under phosphate-limited conditions [78]. A further protein implicated in adaptation to phosphate limitation is PstS that shows increased expression in *Synechococcus *under phosphate limitation [79]. Both P-SSM2 and P-SSM4 have the gene *pstS *[25]. It is thought that cyanomyoviruses maintain *phoH *and *pstS *to allow their host to allow increased phosphate uptake during infection, although the mechanism of how this occurs is unknown.

#### Non-cyanobacterial genes with unknown function in cyanomyoviruses

There are many genes in cyanomyovirus genomes that are similar to hypothetical genes in their hosts, where the host function is not known. Additionally all phage contain bacterial genes that are not found in their cyanobacterial hosts, but appear to have been acquired from other bacterial hosts, this includes the genes *prnA *and *cobS *which encode tryptophan halogenase and an enzyme that catalyses the final step in cobalamin synthesis respectively. Tryptophan halogenase is not found in any known host of cyanomyoviruses, however it is known to catalyse the first step in the biosynthesis of the fungicide pyrrolnitrin in *Pseudomonas fluorescens *[80]. It has been suggested that it may function to provide antibiotic protection to its host, however as stated by the authors this idea is speculative [23]. It has been suggested that *cobS *may boost the production of cobalamin during phage infection [25], the resulting effect of increased cobalamin levels is not known. Potentially it may increase the activity of ribonucleotide reductases, although if it did the process would be unique to cyanophages [25].

#### Metabolic coup d'etat

Cyanomyoviruses may also affect host metabolism on a far grander scale than simply expressing genes to replace the function of host genes such as *psbA *or *talC*. The gene *mazG *has been found in all cyanomyovirus genomes sequenced to data and has also been found to be widespread in cyanomyovirus isolates [81]. MazG has recently been shown to hydrolyse ppGpp in *E. coli *[82]. ppGpp is known as a global regulator of gene expression in bacteria, it also shows increased expression in cyanobacteria under high-light conditions [83]. It has been proposed that the phage fools its host cell into believing it is in nutrient replete conditions, rather than the nutrient deplete conditions of an oligotrophic environment where *Synechococcus *and *Prochlorococcus *dominate [11]. It is thought to do this by the reducing the pool of ppGpp in the host which regulates global gene expression causing the host to modify its physiological state for optimal macromolecular synthesis thus most favourable conditions for production of progeny phage [84].

#### Gene transfer between the T4-likes and their hosts (impact on host genome evolution in the microbial world)

As discussed in the preceding sections there is clear evidence that cyanophages have acquired a plethora of genes from their bacterial hosts. These are recognisable either by being highly conserved such as *psbA *which is conserved the amino acid level, or by the presence of a shared conserved domain with a known gene. Phages potentially have two methods of donating phage genes back to their hosts; through generalised or specialised transduction. Generalised transduction results from non-productive infections where phages accidently package a head full of host DNA during the stage when their heads are being packaged and they inject this into a second host cell during a non-fatal infection. Specialised transduction in comparison results from the accidental acquisition of a host gene resulting from imprecise excision from a host which would occur during lysogenic induction. Although this area has been poorly studied there is some evidence for both generalised and specialised transduction in cyanophages [85].

Despite little direct evidence of lysogeny in marine cyanophages the relationship between host and phage genes can be established from phylogenetic analyses. When host genes are acquired by phages, they generally drift from having the GC composition of their hosts to that of the phage genome. This difference is much clearer in *Synechococcus*-phage relationships because *Synechococcus *genomes have a GC % of around 60% compared to the phages which have a GC% of around 40%. The GC of *psbA *in *Synechococcus *phages has drifted to a value between the average host and phage GC% so is around 50%. These differences are less clear in *Prochlorococcus *as it tends to have a similar CG% to the phages which infect it and thus phylogenetic analysis can be dominated by homoplasies (the same mutation happening independently).

All of the robust phylogenetic analyses that have been performed on metabolic phage genes that are shared between hosts and phages suggest that phages have generally picked up host genes on limited occasions and this has been followed by radiation has within the phage populations for example see Millard et al. 2005 [53].

There is nothing known about the biology and molecular basis of lysogeny or pseudolysogeny in T4 type cyanomyoviruses. Indirect evidence for the abundance of lysogens was obtained from studies on inducing wild populations of cyanobacteria and quantifying the number of potential phages using epifluorescence. This work demonstrated that more temperate phages could be induced in winter when the number of cyanobacterial hosts was low and so conditions were hostile for phages in the lytic part of their life cycle. Other studies have suggested that the apparent resistance *Synechococcus *shows to viral infection may be due to lysogenic infection [3]. It is also clear that the phosphate status of cyanobacteria influences the dynamics of integration [86]. During nutrient starvation cyanoviruses enter their hosts but do not lyse the cells, their genes are expressed during this period (Clokie et al., unpublished). The cells are lysed when phosphate is added back into the media. It is not known exactly how cyanophage DNA is integrated into the cell during this psuedolysogenic period but this may be a time in which genes may be donated and integrated from the phage genome to that of the host.

Despite a lack direct evidence for phage-mediated gene transfer, it is likely that transduction is a major driver in cyanobacterial evolution as the other methods of evolution are not available to them. In the open oceans DNA is present at such low levels (0.6 - 88 μg liter^-1^) that it is probably too dilute for frequent transformation [87]. Also both *Synechococcus *and *Prochlorococcus *appear to lack plasmids and transposons rendering conjugation an unlikely method for the acquisition of new genes. The large number of bacteriophages present in the oceans as well as the observation that phage-like particles appear to be induced from marine cyanobacteria, along with phage-like genes found in cyanobacterial genomes suggests that transduction is evident as a mechanism of evolution.

The genetic advantages that the T4-like cyanomyoviruses may confer to their hosts were listed in a recent review, but in brief they are: (1) prophages may function as transposons, essentially acting as foci for gene rearrangements, (2) they may interrupt genes through silencing non-essential gene functions, (3) they may confer resistance to infection from other phages, (4) they may excise and kill closely related strains, (5) they may cause increased fitness by the presence of physiologically important genes or (6) the phages may silence host genes.

In summary, it is difficult to pin down the exact contribution that T4-like cyanoviruses play in microbial evolution but their abundance, modes of infection and genetic content imply that they may be extremely important for cyanobacterial evolution. Their contribution will become clearer as more genomes are sequenced and as genetic systems are developed to experiment with model systems.

#### The impact of cyanomyoviruses on host populations

The two major biotic causes of bacterial mortality in the marine environment are phage-induced lysis and protistan grazing, currently efforts are being made to assess the relative impacts of these two processes on marine cyanobacterial communities. Accurate information is difficult to obtain for the oligotrophic oceans because of intrinsically slow rate processes [88]. It must also be borne in mind that there are likely to be extensive interactions between the two processes e.g. phage-infected cells might less or more attractive to grazers, phage-infected cells might be less or more resistant to digestion in the food vacuole and phages themselves might be subject to grazing. Estimates of the relative effects of phage-induced lysis and grazing on marine cyanobacterial assemblages vary widely e.g. [89-91] and this probably reflects the fact the two processes do vary widely on both temporal and spatial scales.

A number of methods have been developed to assess viral activity in aquatic systems, but all suffer from a variety of limitations such as extensive sample manipulation or poorly constrained assumptions [92,93]. The application of these approaches to studying cyanomyovirus impact on *Synechococcus *populations has produced widely varying results. Waterbury and Valois [3] calculated that between 0.005% (at the end of the spring bloom) and 3.2% (during a *Synechococcus *peak in July) of the *Synechococcus *population was infected on a daily basis. Another study [94] indicated that as many as 33% of the *Synechococcus *population would have to have been lysed daily at one of the sampling stations. A subsequent study using the same approach [95] yielded figures for the proportion of the *Synechococcus *community infected ranging from 1 - 8% for offshore waters, but in nearshore waters only 0.01 - 0.02% were lysed on a daily basis. Proctor and Fuhrman [96] found that, depending on the sampling station, between 0.8% and 2.8% of cyanobacterial cells contained mature phage virions and making the questionable assumption that phage particles were only visible for 10% of the infection cycle, it was calculated that percentage of infected cells was actually ten-fold greater than the observed frequency.

An important consideration in attempting to establish the impact of cyanomyoviruses on their host populations is to ask at what point the infection rate becomes a significant selection pressure on a population, leading either to the succession of intrinsically resistant strains, or the appearance of resistant mutants. It has been calculated that the threshold would occur between 10^2 ^and 10^4 ^cells ml^-1 ^[10] and this is in agreement with data from natural *Synechococcus *populations that suggest that a genetically homogeneous population would start to experience significant selection pressure when it reached a density of between 10^3 ^and 10^4 ^cells ml^-1 ^[97].

The community ecology of cyanomyovirus-host interactions is complicated by a number of factors including the genetic diversity of phages and hosts, protistan grazing and variations in abiotic factors (e.g. light, nutrients, temperature). Thus simple modelling of predator-prey dynamics is not possible. However, a "kill the winner" model [92,98] in which the best competitor will become subject to infection has gained widespread acceptance. Recently, marine phage metagenomic data have been used to test theoretical models of phage communities [99] and the rank-abundance curve for marine phage communities is consistent with a power law distribution in which the dominant phage keeps changing and in which host ecotypes at very low numbers evade phage predation. A variety of studies have looked at spatio-temporal variations in cyanomyovirus populations. The earliest studies showed that cyanomyovirus abundance changed through an annual cycle [3] and with distance from shore, season and depth [94]. The ability to look at the diversity of cyanomyovirus population using *g20 *primers revealed that maximum diversity in a stratified water column was correlated with maximum *Synechococcus *population density [30] and changes in phage clonal diversity were observed from the surface water down to the deep chlorophyll maximum in the open ocean [28]. Marston and Sallee [35] found temporal changes in both the abundance, overall composition of the cyanophage community and the relative abundance of specific *g20 *genotypes In Rhode Island's coastal waters. Sandaa and Larsen [34] also observed seasonal variations in the abundance of cyanophages and in cyanomyovirus community composition in Norwegian coastal waters. Cyanomyovirus abundance and depth distribution was monitored over an annual cycle in the Gulf of Aqaba [40]. Cyanophages were found throughout the water column to a depth of 150 m, with a discrete maximum in the summer months and at a depth of 30 m. Whilst it is clear from all these studies that cyanomyovirus abundance and community composition changes on both a seasonal and spatial basis, little is know about short term variations. However, one study in the Indian Ocean showed that phage abundance peaked at around 0100 at a depth of 10 m, but the temporal variation was not as strong at greater depths [84]. It may well be the case that infection by cyanomyoviruses is a diel phenomenon as phage adsorption to host is light-dependent for several marine cyanomyoviruses studied [100]. A similar observation for the freshwater cyanomyovirus AS-1 [101]. There is currently only one published study that describes attempts to look at the co-variation in the composition of *Synechococcus *and cyanomyovirus communities to establish whether they were co-dependent [102]. In the Gulf of Aqaba, Red Sea, a succession of *Synechococcus *genotypes was observed over an annual cycle. There were large changes in the genetic diversity of *Synechococcus *, as determined by RFLP analysis of a 403 bp *rpoC1 *gene fragment, which was reduced to one dominant genotype in July. The abundance of co-occurring cyanophages capable of infecting marine *Synechococcus *was determined by plaque assays and their genetic diversity was determined by denaturing gradient gel electrophoresis analysis of a 118 bp *g20 *gene fragment. The results indicate that both abundance and genetic diversity of cyanophage covaried with that of *Synechococcus*. Multivariate statistical analyses show a significant relationship between cyanophage assemblage structure and that of *Synechococcus*. All these observations are consistent with cyanophage infection being a major controlling factor in cyanobacterial diversity and succession.

Analysis of the impact of cyanomyoviruses on host populations has been based on the assumption that they follow the conventional infection, replication and cell lysis life cycle, but there is some evidence to suggest that this may not always be the case. There is one particularly controversial area of phage biology and that is the topic of pseudolysogeny. There are in fact a variety of definitions of pseudolysogeny in the literature reflecting some quite different aspects of phage life history, but the one adopted here is "the presence of a temporarily non-replicating phage genome (a preprophage) within a poorly replicating bacterium" (S. Abedon - personal communication). The cyanobacterial hosts exist in an extremely oligotrophic environment posing constant nutritional stress and are exposed to additional environmental challenges such as light stress that may lead to rates of growth and replication that are far from maximal. There is evidence that obligately lytic *Synechococcus *phages can enter such a pseudolysogenic state. When phage S-PM2 (a myovirus) was used to infect *Synechococcus *sp. WH7803 cells grown in phosphate-replete or phosphate-deplete media there was no change in the adsorption rate constant, but there was an apparent 80% reduction in the burst size under phosphate-deplete conditions and similar observations were made with two other obligately lytic *Synechococcus *myoviruses, S-WHM1 and S-BM1 [86]. However, a more detailed analysis revealed this was due to a reduction in the proportion of cells lysing. 100% of the phosphate-replete cells lysed, compared to only 9% of the phosphate-deplete cells, suggesting that the majority of phosphate deplete cells were pseudolysogens.

From very early on in the study of marine cyanomyoviruses it was recognized that phage-resistance was likely to be an important feature of the dynamics of phage-host interactions. Waterbury and Valois [3] found that coastal *Synechococcus *strains were resistant to their co-occurring phages and suggested that the phage population was maintained by a small proportion of cells sensitive to infection. For well studied phage-host systems resistance is most commonly achieved by mutational loss of phage receptor on the surface of the cell, though there are other mechanisms of resistance to phage infection e.g. [103]. Stoddard et al. [104] used a combination of 32 genetically distinct cyanomyoviruses and four host strains to isolate phage-resistant mutants. Characterization of the mutants indicated that resistance was most likely due to loss or modification of receptor structures. Frequently, acquisition of resistance to one phage led to cross-resistance to one or more other phages. It is thought that mutation to phage resistance may frequently involve a fitness cost and this trade-off allows the coexistence of more competitive phage-sensitive and less competitive phage-resistant strains (for review see [105]). The cost of phage resistance in marine cyanobacteria has been investigated by Lennon et al. [106] using phylogenetically distinct *Synechococcus *strains and phage-resistant mutants derived from them. Two approaches were used to assess the cost of resistance (COR); measurement of alterations in maximum growth rate and competition experiments. A COR was found in roughly 50% of cases and when detected resulted in a ~20% reduction in relative fitness. Competition experiments suggested that fitness costs were associated with the acquisition of resistance to particular phages. A COR might be expected to be more clearly observed when strains are growing in their natural oligotrophic environment. The acquisition of resistance to one particular cyanophage, S-PM2, is associated with a change in the structure of the lipopolysaccharide (LPS) (E. Spence - personal communication).

A variety of observations arising from genomic sequencing have emphasized the role of alterations in the cell envelope in the speciation *Prochlorococcus *and *Synechococcus *strains, presumably as a result of selection pressures arising from phage infection or protistan grazing. An analysis of 12 *Prochlorococcus *genomes [107] revealed a number of highly variable genomic islands containing many of the strain-specific genes. Amongst these genes the greatest differentiator between the most closely related isolates were genes related to outer membrane synthesis such as acyltransferases. Similar genomic islands, containing the majority of strain-specific genes, were identified through an analysis of the genomes of 11 *Synechococcus *strains [108]. Among the island genes with known function the predominant group were those encoding glycosyl transferases and glycoside hydrolases potentially involved in outer membrane/cell wall biogenesis. The cyanomyovirus P-SSM2 was found to contain 24 LPS genes that form two major clusters [25]. It was suggested that these LPS genes might be involved in altering the cell surface composition of the infected host during pseudolysogeny to prevent infection by other phages. The same idea could apply to a normal lytic infection and could be extended to protection against protistan grazing. Similarly, cyanomyovirus S-PM2 encodes a protein with an S-layer homology domain. S-layers are quasi-crystalline layers on the bacterial cell surface and so this protein, known to be expressed in the infected cell as one of the earliest and most abundantly transcribed genes [56], may have a protective function against infection or grazing.

#### The potential value of continuing research on the "eco-genomics" of cyanophages

Eco-genomics is defined as the application of molecular techniques to ecology whereby biodiversity is considered at the DNA level and this knowledge is then used to understand the ecology and evolutionary processes of ecosystems. Cyanophage genomes encode a huge body of unexplored biodiversity which needs to be understood to further extend our knowledge of cyanophage-cyanobacteria interactions and thus to fully appreciate the multiple roles that cyanophages play in influencing bacterial evolution, physiology and biogeochemical cycling.

As cyanophage genomes are stripped down versions of essential gene combinations an understanding of their genomics will assist in defining key host genes that are essential for phage reproduction. As many of the host genes encoded in phage genomes have an unknown function in their hosts, the study of phage genomes will impinge positively on our understanding of cyanobacterial genomes. The other major spin-off from researching the products encoded by phage genomes is the discovery of novel enzymes or alternative versions existing enzymes with novel substrate specificities. This is likely to be of major importance to the biotechnology and pharmaceutical industries.

As more phage genomes and metagenomes are sequenced, the core set of phage genes will be refined and the extent of phage encoded host metabolic and other accessory genes will be revealed. We would expect to find specific environments selecting particular types of genes. This research area is often referred to as 'fishing expeditions' especially by grant panels. However it is analogous to the great collections of plants and animals that occurred during the 19^th ^Century. These data were collected over a long period of time and it was only subsequently that scientists understood patterns of evolution, biogeography, variance and dispersal. This is an exciting time to be mining cyanophage genomes as metagenomic analysis of the viral fraction from marine ecosystems has suggested that there is little restriction to the types of genes that bacteriophages can carry [109]. These data will likely provide the bedrock on which generations of scientists can interpret and make sense of.

To drive our understanding of cyanophage genomes forward however there needs to a concerted effort to capitalise on the sequence libraries that are being collected from both phage metagenomes and phage genomes. Sequencing even large data-sets is now comparatively easy and sequence information should be seen as the exciting starting point rather than the endpoint. To determine the function of the reservoir of genes will require extensive biochemical, chemical and molecular biological investigations as well as physiological experimentation.

Currently when new T4-like cyanophage genes are identified using bioinformatic approaches, they are compared to T4 and their function is deduced on the basis of known genes in the T4 genome. In order to really progress with understanding the role of the genes which have no homology (and to confirm the homology in genes where an identity can be hypothesised) a genetic system needs to be developed where cyanophages can be mutated. This will take extensive research effort and hopefully international research groups will come together in the way that researchers on the T phages in the 1960 s did to gradually piece together to determine the function of the genes that constitute largest reservoir of genetic diversity on earth.

## Conclusions

The study of the "photosynthetic" cyanomyoviruses has revealed novel and important facets of the phage-host relationship that were not apparent from previous studies with heterotrophic systems. However, in common with all the T4-like phages there is much work to do in ascribing functions to the many genes lacking known homologues. It is probable that many of these genes are involved in the subtle manipulation of the physiology of the infected cell and are likely to be of potential importance in biotechnology as well as being intrinsically interesting. However, there are three main features specific to marine cyanomyovirus biology that require further substantial attention. At present there has been little more than speculation and theoretical modelling on the contribution of host-derived genes to cyanomyovirus fitness and it is important to develop experimental approaches that will enable us to assess the contribution the genes make to the infection process. There is also the related topic of evaluating the role of these phages as agents of horizontal gene transfer and assessing their contribution to cyanobacterial adaptation and evolution. Furthermore, from the ecological perspective we are still a long way from being able to assess the true impact of these cyanomyoviruses on natural populations of their hosts. It is likely that these cyanomyoviruses will remain an important feature of research in both phage biology and marine ecology for a considerable while to come.

## Abbreviations

PBPs: phycobilin-bearing phycobiliproteins; APC: allophycocyanin; PC: phycocyanin; PE: phycoerytherin; Chl a: chlorophyll a; nm: nanometer; GOS: global ocean sampling; Q-PCR: quantitative polymerase chain reaction; nr: non redundant; ORF(s): open reading frame(s); LPS: lipopolyscacchride; PSII: photosystem II.

## Competing interests

The authors declare that they have no competing interests.

## Authors' contributions

ADM, MRJC & NHM contributed to the original drafts of the manuscript and approved the final version.
